# Back-propagation optimization and multi-valued artificial neural networks for highly vivid structural color filter metasurfaces

**DOI:** 10.1038/s41598-023-48064-x

**Published:** 2023-12-04

**Authors:** Arthur Clini de Souza, Stéphane Lanteri, Hugo Enrique Hernández-Figueroa, Marco Abbarchi, David Grosso, Badre Kerzabi, Mahmoud Elsawy

**Affiliations:** 1 Université Côte d’Azur, Inria, CNRS, LJAD, 06902 Sophia Antipolis Cedex, France; 2 Laboratory of Applied and Computational Electromagnetism (LEMAC), School of Electrical and Computer Engineering (FEEC), University of Campinas (UNICAMP) Campinas, São Paulo, Brazil; 3 Solnil, 95 Rue de la République, 13002 Marseille, France; 4 Université Aix Marseille, CNRS, Université de Toulon, IM2NP, UMR 7334, F-13397 Marseille, France

**Keywords:** Engineering, Materials science, Mathematics and computing, Optics and photonics

## Abstract

We introduce a novel technique for designing color filter metasurfaces using a data-driven approach based on deep learning. Our innovative approach employs inverse design principles to identify highly efficient designs that outperform all the configurations in the dataset, which consists of 585 distinct geometries solely. By combining Multi-Valued Artificial Neural Networks and back-propagation optimization, we overcome the limitations of previous approaches, such as poor performance due to extrapolation and undesired local minima. Consequently, we successfully create reliable and highly efficient configurations for metasurface color filters capable of producing exceptionally vivid colors that go beyond the sRGB gamut. Furthermore, our deep learning technique can be extended to design various pixellated metasurface configurations with different functionalities.

## Introduction

Optical color filters are structures or materials designed to discriminate and manipulate distinct light wavelengths through the selective transmission or reflection of particular colors while simultaneously absorbing or attenuating undesired colors^1,2^. Conventional color filters rely on the manipulation of chemical composition to achieve the desired optical properties, which can lead to issues such as absorption losses, thermal effects, and alterations in chemical characteristics^3^. An alternative approach involves the utilisation of structural color filters, offering distinct advantages and applications in diverse fields such as photorealistic color printing, color holography, anti-counterfeiting devices, and much more^4–6^.

Metasurfaces have emerged as a promising platform for structural color filters^7,8^, owing to its peculiar capability of controlling all the light properties at the nanoscale, enabling a plethora of applications^9–12^. Dielectric metasurfaces play a crucial role in color filter applications, especially within the visible spectrum range where the plasmonic conterpart based on metals is less performing owing to intrinsic optical losses. The limited losses of dielectrics (e.g. Si_3_N_4_, GaN, TiO_2_, ZrO_2_, HfO_2_) make them highly desirable for designing efficient devices with sharp resonance responses^13–16^. Resonant dielectric metasurfaces achieve precise control over the phase of reflected and transmitted light by leveraging various resonant phenomena (e.g. Mie resonances)^17,18^. Through meticulous engineering of the resonators, selective interaction with different wavelengths is enabled, leading to efficient and vivid color filters. Such kind of metasurfaces offer exceptional phase control, high-quality factors, and sharp resonances, resulting in enhanced color purity and spectral selectivity^19–21^. Yet, the design of an ideal color filter demands capability to selectively filter all colors across the optical spectrum. In other words, at each desired wavelength, it is crucial to eliminate any background resonances in order to achieve a pure color response characterized by sharp reflection or transmission amplitudes. Given the fabrication constraints, finding the appropriate resonator shape to achieve a desired response, is a challenging task that has garnered significant attention in the research community. Numerous studies explored this area, employing sophisticated optimization algorithms including advanced Deep Learning (DL) approaches to tackle the inherent complexity of the problem^20–29^. However, relying on classical optimization approaches requires several costly simulations when optimizing various color targets simultaneously^30–32^. A viable solution for the design of vivid metasurface color filters is one-shot optimization using Artificial Neural Network (ANN). However, it is not straightforward owing to the presence of several designs with similar optical response whereas regular ANN has only a single output^33^.

Here we present a novel data-driven methodology for efficiently designing fabrication constrained color filter metasurfaces. Our approach combines the ability to find suitable designs of Multi-Valued Artificial Neural Network (MVANN) with the solution refinement of back-propagation optimization. Thereby overcoming the fundamental limitations of relying on either the latter, which will lead to an undesirable local minima, or solely on a MVANN leading to poor performance associated with extrapolation^23,24,33^. In our case, relying solely on 585 simulations and by varying four parameters allows for the optimization of a continuous spectrum of objectives and the identification of highly vivid metasurface color filters. The optimized geometries exhibit a single sharp resonance response representing all the colors across the visible regime. To the best of our knowledge, our research outcomes exceed the previous findings documented in the literature, positioning our color filter as the foremost advancement in terms of vividness and overall performance^8,29,34–36^.

## Geometry and surrogate model

**Figure 1:**
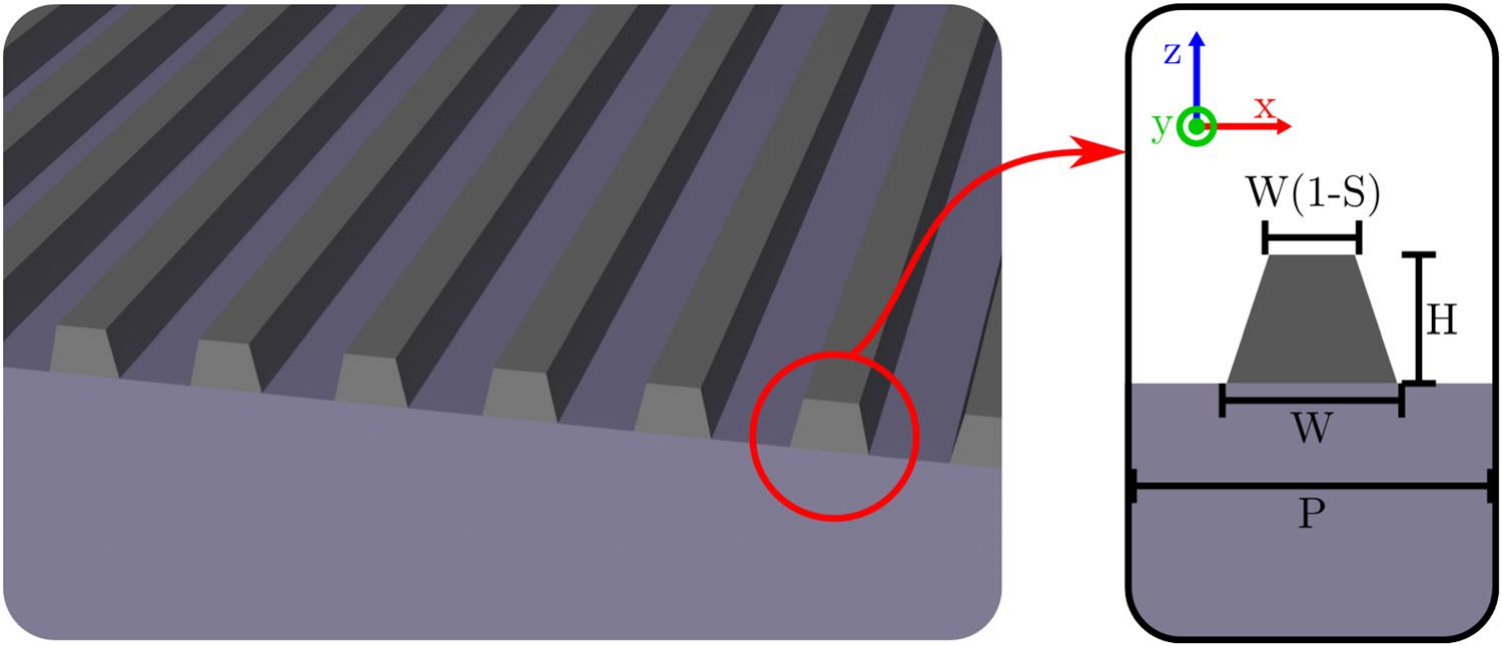
Schematic representation of the simulated structure. The light is injected from top with normal incidence with electric field polarized along the x-direction. The inset refers to the geometrical parameters associated to the single unit-cell surrounded with periodic boundary conditions.

Figure 1 represents the considered metasurface geometry composed of slanted ridges of titanium dioxide (TiO_2_) on top of a silicon dioxide (SiO_2_) substrate with refractive index $\mathrm {n_s = 1.45}$. The refractive index of TiO_2_ is determined through ellipsometry (available in the supplementary information section). The inclusion of slanted gratings in the metasurface design introduces additional degrees of freedom, enabling the appearance of sharp resonances in the reflection spectrum. This is achieved by breaking the symmetry in the z-direction, resulting in high-quality resonance modes^37,38^. Four parameters, namely the metasurface period (P), resonator height (H), base width (W), and the ratio between the top and base widths of the resonator (S), are optimized in this study.

The first step to train the ANN is to generate a dataset. One important aspect that we considered to build it is the fabrication constraint that imposes a maximum aspect ratio of 2 between the height and width of the resonators. The dataset consists of 585 simulations generated by uniformly sweeping the four parameters indicated in Fig. 1. The period P ranges from 250 and 510 nm. For the height H, the range spans from 100 to 425 nm. In order to ensure that the ratios H/W and H/(P-W) remain less than 2, the width W varies between H/2 and (2P-H)/2, and therefore, the height H must obey the inequality H/2 < (2P-H)/2. Additionally, the parameter S ranges from 0 to 0.8, with intervals of 0.1. The simulations were performed on an Intel$\circledR$ Xeon$\circledR$ W-2125 Processor operating at 4.0 GHz and allocating 4 threads, taking a total of 33 min and 40 s. The description of the 2D Python MEEP Finite-Difference Time-Domain (FDTD) simulations are described in the first section in the supplementary information.

Subsequently, a feedforward ANN surrogate model was trained to forecast the reflection spectral response, considering the resonator’s geometry as input. In this study, we leverage the surrogate model for two distinct purposes. Firstly, it enables rapid estimation of the reflection spectrum, achieving significant computational speed-ups compared to full wave FDTD simulations. Secondly, the surrogate model is crucial in the inverse design process, playing a critical role in computing optimal solutions. As a result, the surrogate model’s performance is of paramount importance, as it must deliver exceptional precision to provide the most accurate approximations possible. By fulfilling these requirements, the surrogate model accuracy significantly contributes to the effectiveness and success of this study. Our surrogate model is a classical Multilayer Perceptron (MLP) model with fully connected layers. In this scenario, there are several ways to configure this MLP, and we will compare two different strategies to map the input geometries into a spectrum.

**Figure 2:**
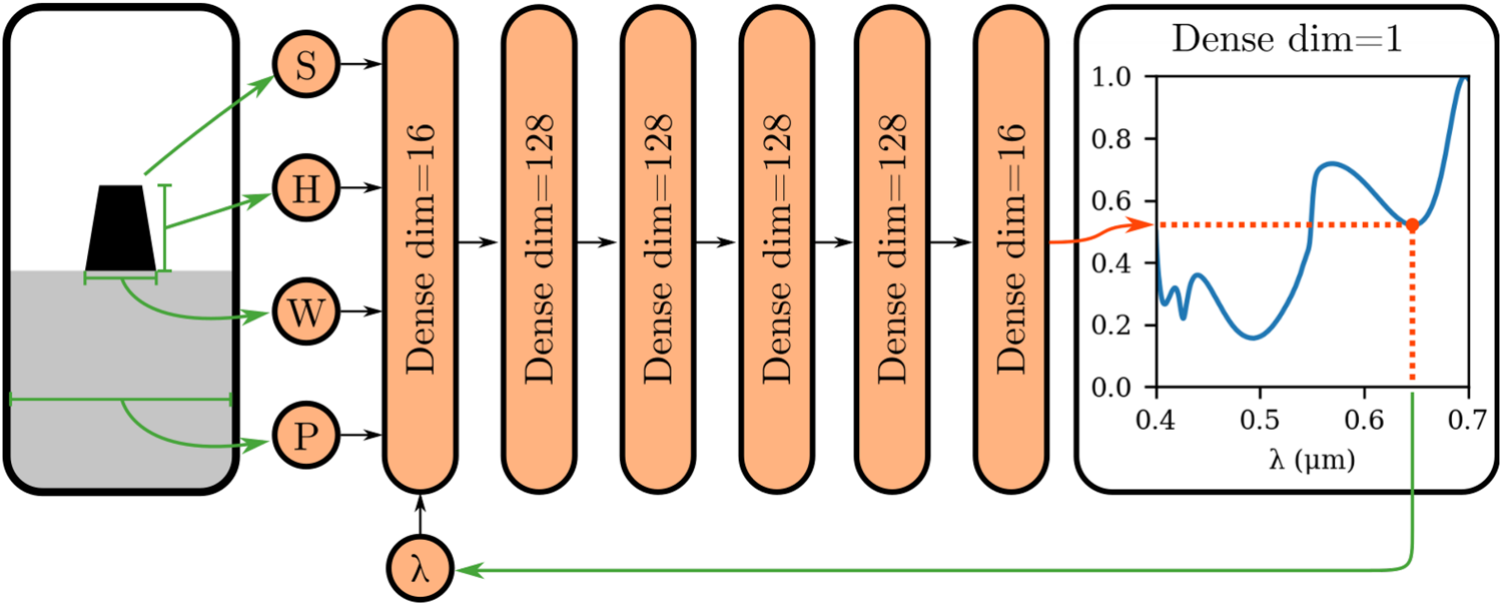
The diagram represents the architecture of the surrogate model ANN. The green arrows represent the input parameters. The surrogate model is designed to calculate a single output dimension, which corresponds to the reflection at the desired wavelength as shown by the red arrow. On the left side, a typical example of the resonator’s geometry is provided, while on the right side, an example spectrum is displayed. It is important to note that these examples are purely for illustrative purposes and do not represent any real simulations. All the layers in this architecture are initialized using Glorot normal initializers as discussed in Ref.^40^. The model was trained using the TensorFlow framework, employing a holdout methodology^41^. The dataset is divided into two subsets: 555 simulations to update the weights of the ANN and 30 simulations for validation. The optimizer of choice was Adam with Mean Square Error (MSE) loss and batch size of 512^42^. The training stopped at epoch 82 due to the lack of improvement of the validation loss within the last 20 epochs. The final MSE loss was $2.6996\times 10^{-4}$ and validation loss, $3.2745\times 10^{-4}$. The graph showing the loss evolution at each epoch can be found in section 2 of the supplementary information document. The total training time was 9 min 48s on a Google colab’s Central Processing Unit (CPU).

The first approach uses the geometric parameters as inputs and generates a multi-dimensional vector as output. Each dimension of this vector corresponds to a distinct wavelength in the reflection spectrum. The second approach involves incorporating the wavelength as an input parameter while outputting a single dimension representing the reflection amplitude at that particular wavelength (Fig. 2). Although both methods initially seems to be identical, the second is more convenient for this study. By incorporating the wavelength as an input, each point in the spectrum becomes a unique data point for the surrogate model. In contrast, the first method contains only 585 data points available for training the ANN. Conversely, the second method significantly enhances the availability of data samples, yielding a total of 292,500 individual data points resulting from sampling the spectrum across 500 different wavelengths.

Figure 2 represents the architecture of the surrogate model. In general, we consider the Gaussian Error Linear Unit (GELU) activation function which is a gating activation with a continuous derivative. Considering GELU implies the continuity of the output and its derivative, providing smoother response compared to the classical Rectified Linear Unit (ReLU) activation^39^. It is worth mentioning that the last layer contains a single linear neuron to compute a singular output dimension aligning with the reflection observed at the designated wavelength as indicated by the red arrow in Fig. 2.

**Figure 3:**
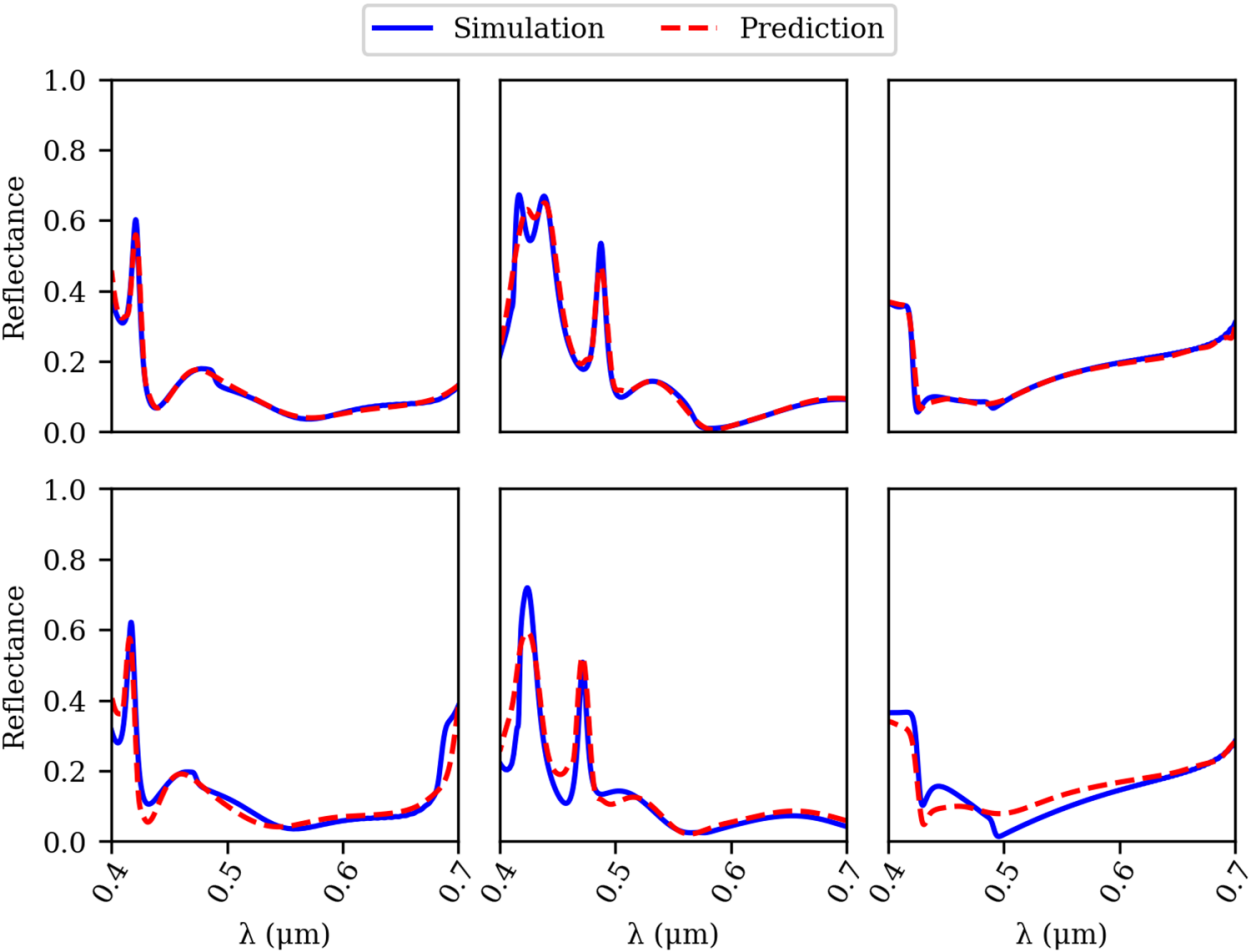
Comparison between fullwave simulation and the prediction from the surrogate model. Top figures indicate the reflection spectra of three randomly selected samples from the dataset. The bottom figures corresponding to the same top geometries while introducing a small random deviation to the geometrical parameters. Notably, the surrogate model demonstrates robust generalization capabilities despite the inherent sparsity of the dataset.

Figure 3 illustrates the generalization capacity of the surrogate model. Despite being trained on a relatively small dataset, the model is able to reproduce the spectra associated to the geometries that lie beyond the training dataset. This accomplishment can be attributed to the integration of wavelength as an input variable and the meticulous training methodology employed to mitigate overfitting. The demonstrated efficiency of this approach underscores the potential for constructing dependable surrogate models even when confronted with restricted training data.

## Inverse design

In this particular section, an inverse design methodology is formulated for metasurface color filter. The primary objective is to determine the corresponding set of parameters based on the desired spectrum response. To initiate the process, the target line shape spectrum must be established, and in our research, a Lorentzian function shape resembling a Fano resonance-like shape is employed:

1 $$\begin{aligned} L\left( f\right) = \frac{\omega ^2}{\omega ^2 + 4\left( f - f_0\right) ^2}, \end{aligned}$$

where $\omega$ represents the full width at half maximum (FWHM) while $f_0$ corresponds to the central frequency. The units employed are in accordance with MEEP’s configuration, where both frequency and FWHM are measured in $\upmu {\textrm{m}}^{-1}$. Additionally, it is worth noting that throughout the optimization process, the FWHM remains unchanged for all targets. This means that the Quality Factor (QF) of the desired spectra undergoes variations as a function of $f_0$, in accordance with Eq. (2).

2 $$\begin{aligned} QF:= \frac{f_0}{\omega } \end{aligned}$$

**Figure 4:**
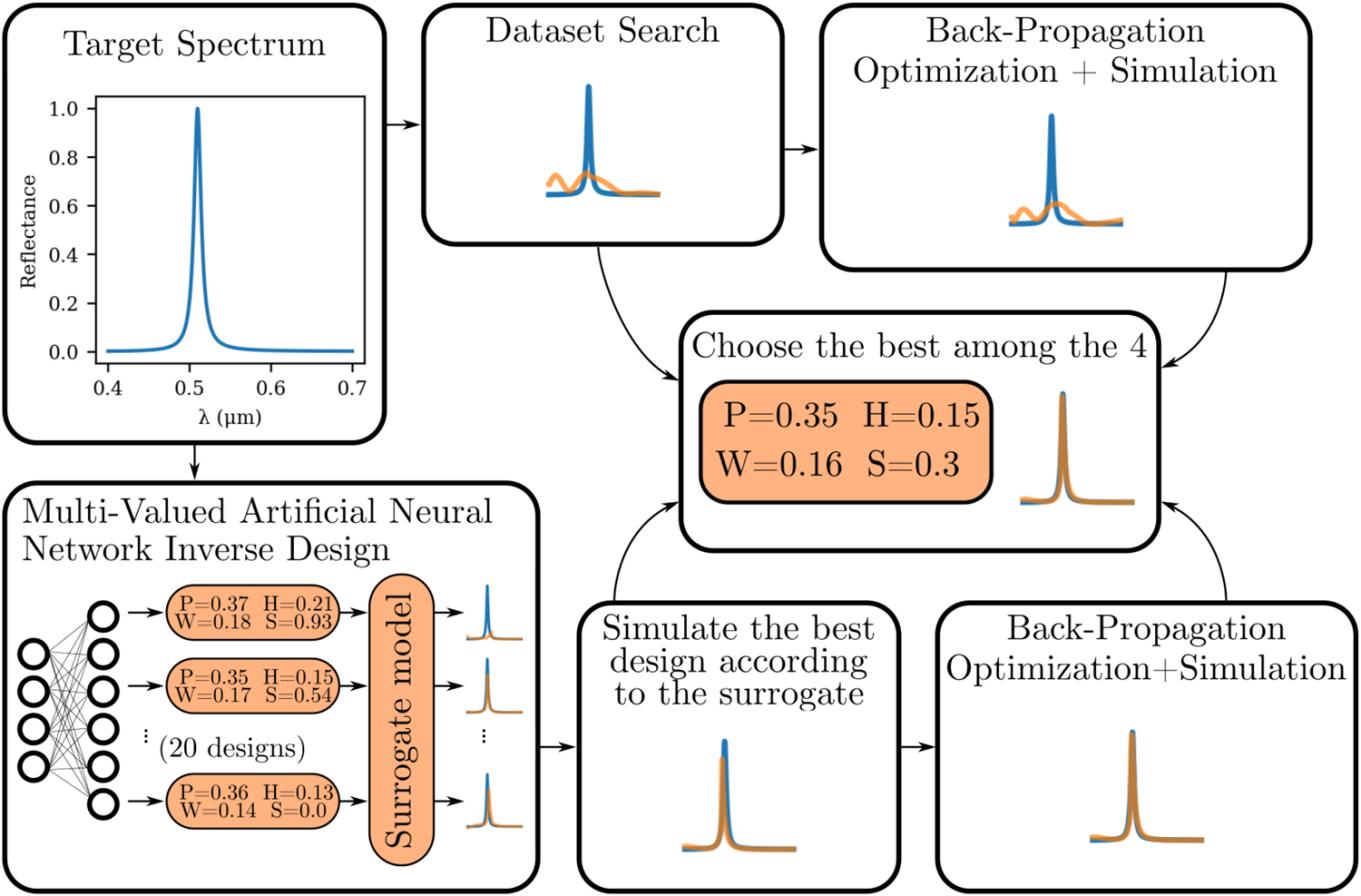
Optimization diagram for the inverse design methodology, where the target reflection spectrum is specified in the cyan curve. To achieve the desired outcome, two complementary paths are identified. The first path involves exploiting the dataset scan and searching for the best design that will be subjected to a back-propagation optimization to fine-tune the parameters, as indicated in the top blocks. On the other hand, the second path involves passing the target through the MVANN to obtain 20 designs. These designs are then validated using the surrogate model, and the solution that presented the least MSE compared to the target is chosen. Finally, a back-propagation optimization is performed on that design. Ultimately, the best solution among the four selected designs from the two paths is chosen. Further details can be found in the text.

**Figure 5:**
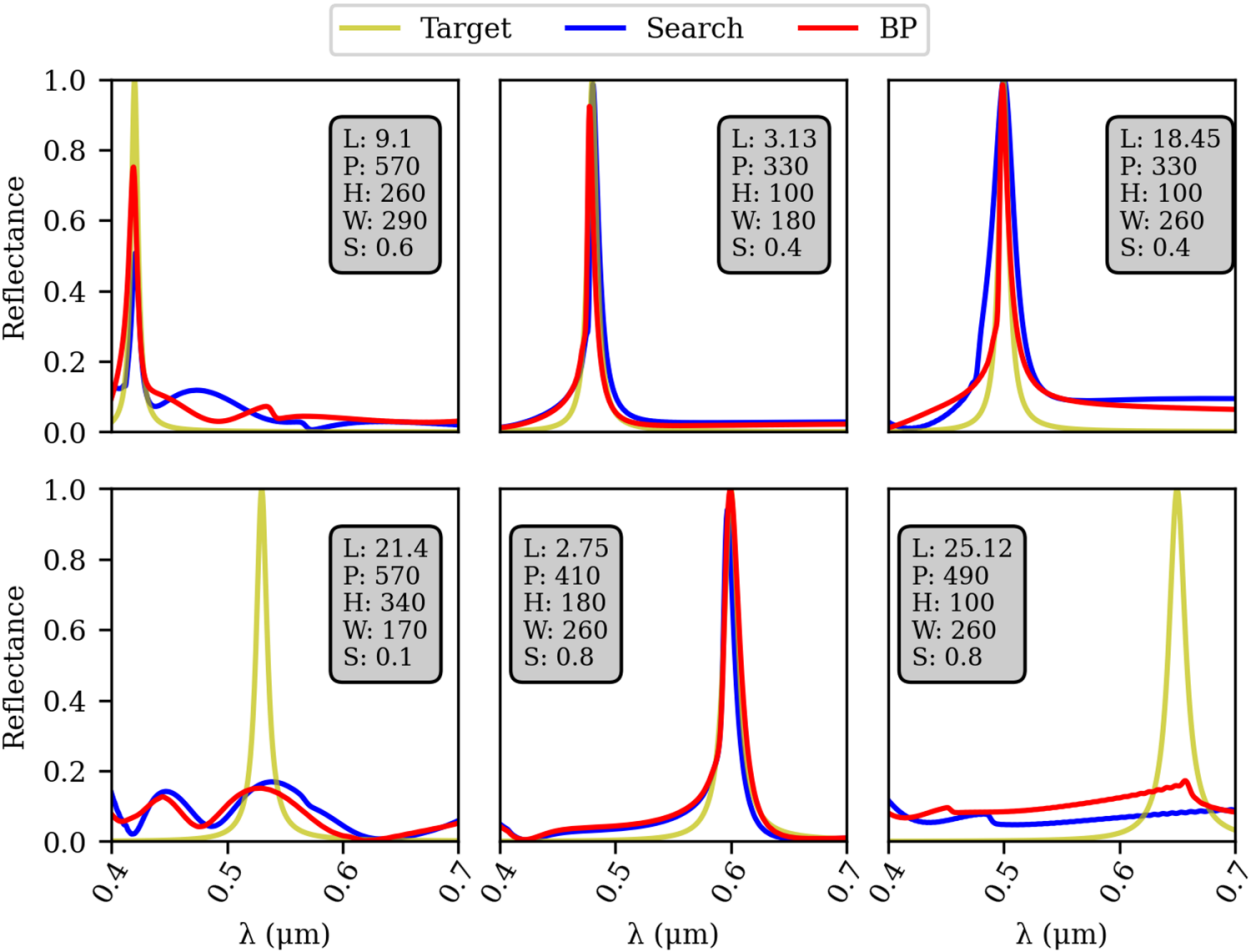
Results from the dataset search using a Lorentzian lineshape in frequency as target for the dataset search for the wavelengths of $420\,\hbox {nm},\,{480}\,{\hbox {nm}}$, ${500}\,{\hbox {nm}}$, ${530}\,{\hbox {nm}}$, ${650}\,{\hbox {nm}},\, {650}\,{\hbox {nm}}$ with $\omega =0.01$ from left to right, top to bottom. The metric used to evaluate the results was the MSE (L) in $10^{-3}$ between the target spectrum and the closest one in the dataset, while the geometric parameters (P, H, W and S) of the device found on the search were described in Fig. 1. Blue and red curves represent the best design without and with back-propagation optimization on best design found in the dataset. A comparison of two different resonance shapes with different $\omega$ values are also tested in figure S5 provided in the supplementary information.

The optimization scenario illustrated in Fig. 4 depicts the inverse design process. The desired spectrum is represented by the cyan curve in the top left corner. In this stage, we employ two complementary approaches for inverse design. The first approach involves a straightforward search through a dataset, represented by the arrow pointing to the right of the target spectrum. For each simulation in the dataset, we calculate the MSE loss between the target spectrum and the simulated spectrum. By doing so, we can identify the design that produces the lowest MSE. Although this design is the best within the dataset, it fails to accurately match the desired response, as indicated in Fig. 5. To address this limitation, we utilize a fully differentiable surrogate model and perform gradient optimization to finely adjust the parameters. As it can be seen in Fig. 5 running the back-propagation yields interesting designs. However, still this approach is not able to produce vivid colors along all the visible regime. Further the resonances are mainly associated with higher order modes appearing at shorter wavelengths (see last column) that allows for non-pure color. The optimized solution, along with the best design found within the dataset, are both stored for later comparison. A detailed explanation of the methodology used to perform back-propagation is displayed in section 3 of the supplementary information.

The second complementary approach, indicated by the downward arrow from the target spectrum in Fig. 4, involves the utilization of a MVANN to generate robust solutions for the inverse design problem. A MVANN is essentially an ANN capable of producing multiple solutions based on a single input. In the context of our study, the adoption of the MVANN proves highly advantageous as it effectively addresses the challenge of multiple parameter responses corresponding to a given objective target^33^. To ensure reasonable accuracy, we choose to employ a model that yields 20 outputs. The detailed architecture and training procedure of the MVANN are provided in the supplementary information section 5. After acquiring the set of 20 distinct designs, a validation process is conducted using the surrogate model. Among these designs, the one that exhibits the lowest MSE loss when compared to the target is initially stored for future comparison. To further improve the results, this best design is subjected to back-propagation optimization. By employing a combination of the dataset search method indicated by the top blocks in Fig. 4 and the MVANN approach, followed by back-propagation indicated by the bottom blocks in Fig. 4, we successfully identify four robust designs. This entire process, encompassing both approaches, takes approximately 100 s to complete. Finally, the design exhibiting the lowest MSE loss compared to the target is selected as the final choice. We refer to section 6 in the supplementary information for more details regarding the performance of MVANN as a function of the dataset size. Further, a detailed comparison between MVANN and a state-of-the art algorithm can be found in section 7 in the supplementary information.

**Figure 6:**
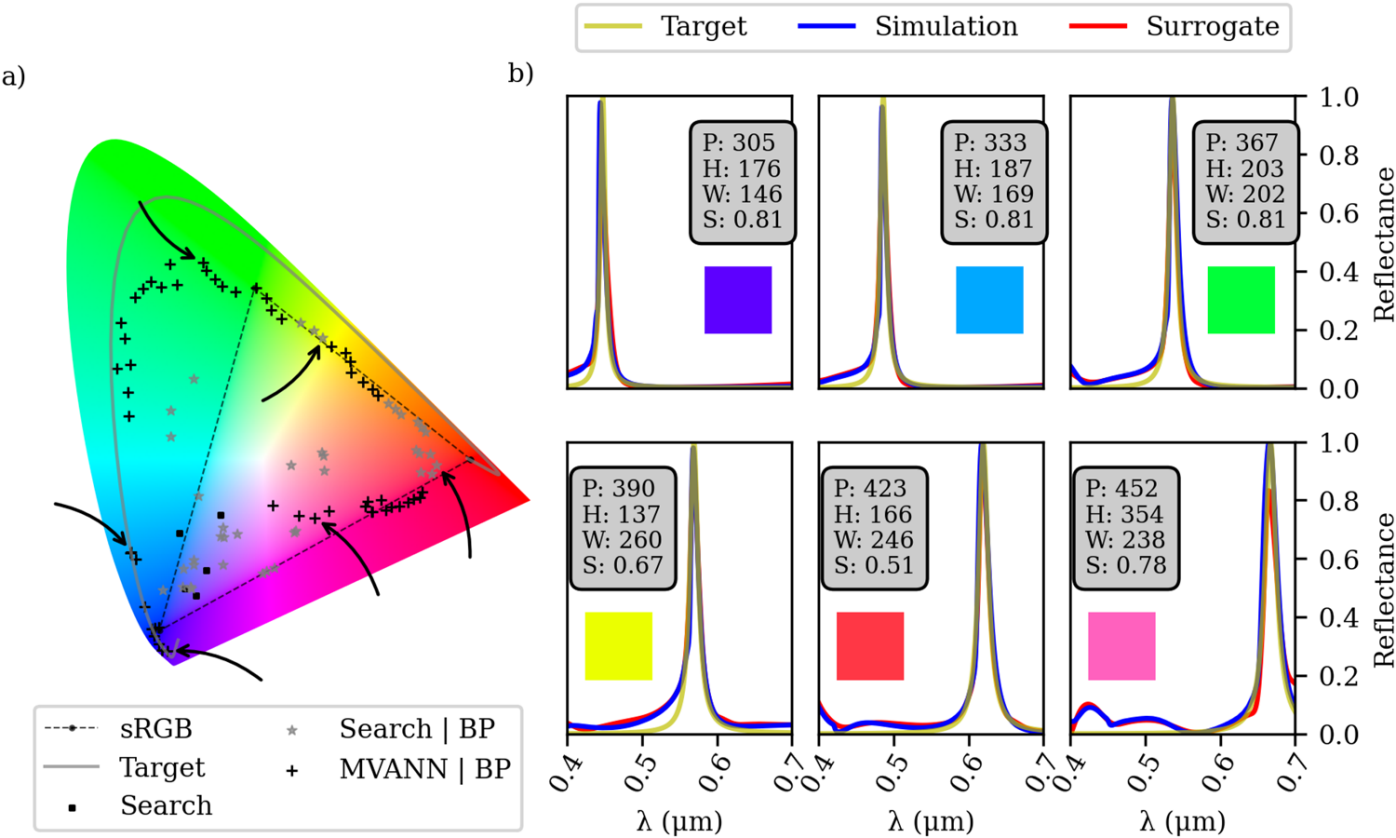
(**a**) Chromaticity diagram depicting the outcomes of the 100 optimization results. The arrows in the diagram indicate the corresponding designs showcased in (**b**). The gray line represents the calculated perceived color corresponding to the target spectrum. (**b**) detailed examination of 6 different optimizations, displaying their dimensions (in nm) of each geometrical parameter and the corresponding sRGB color representation of the simulated spectrum.

By employing the aforementioned inverse design methodology illustrated in Fig. 4, we perform a total of 100 optimization iterations, as depicted in Fig. 6a. These iterations involve sweeping the desired wavelength across the range of 400–700 nm, thereby encompassing the entire spectrum of colors. The target function is defined as a Lorentzian spectrum with a Full Width at Half Maximum (FWHM) value of $\omega = 0.01$. Subsequently, we calculate the perceived color values (x and y) on the chromaticity diagram using the CIE 1931 ${2^{\circ }}$ standard observer color matching functions and the “Equal energy” illuminant, which ensures that incident light of all wavelengths possesses uniform power^43^. As depicted Fig. 6a, we successful optimized metasurface designs that generate colors closely resembling our target (gray curve). Consequently, a significant number of these optimized designs fall outside the sRGB zone, represented by the dashed triangle in Fig. 6a, expanding the color gamut achievable with metasurfaces beyond the limitations of conventional display technologies.

In Fig. 6b, we select six distinct designs to demonstrate the spectral response of our optimized configurations, as indicated by the black arrows in Fig. 6a. Our inverse design approach has led to metasurface designs that exhibit a reflection spectrum response that is consistent with the target Lorentzian spectrum across all desired wavelengths. Moreover, the background response in the spectrum is nearly flat, providing a vibrant color response for our optimized configurations. A typical example of the field profile is presented in the supplementary information. Additionally, a numerical comparison between FDTD calculations and COMSOL has been included to verify our results. Furthermore, we have conducted a full-wave simulation with finite structure to identify the number of required periods to retrive the results of a single unitcell simulation with periodic boundary conditions (for more details, the reader can refer to the supplementary information). To the best of our knowledge, the optimized metasurface designs yield the most vivid color filter reported so far in the literature^8,29,34–36^. It is worth mentioning that the entire optimization process took 2 h, 37 min, and 45 s. Among this time, 1 h, 26 min, and 20 s were dedicated to simulations, 1 h, 2 min, and 6 s were allocated for back-propagation optimization, 8 min and 27 s were spent on predictions using both the surrogate model and the MVANN, and 250 ms were utilized for searching the best design within the dataset.

The results presented in Fig. 6 show that the optimized designs have varying heights. However, considering the complexity associated with fabricating structures of different heights, it is preferable to find designs with a fixed height. Therefore, we introduce a constraint to the back-propagation optimization process, requiring all designs to have equal heights. To achieve this, we modified the inverse design workflow as follows: Initially, we obtained the lowest 5 losses from the MVANN for each of the 6 different target designs, each representing a different color and having a different height. Subsequently, we identified designs that were closer in their height values. We calculated the mean height of these selected designs for each color and used this mean value as an initial guess for optimizing each target using the back-propagation optimization process.

To accommodate the height constraint, we introduced a dedicated layer in the surrogate model. This layer connected the height input solely to the initial guess and consisted of a single weight and bias. This shared layer was employed throughout all optimization iterations. By optimizing all 6 models simultaneously within the same batch, we ensured that the loss for each individual model progressively decreased with each training epoch. Consequently, we transformed the fixed height constraint into an optimized parameter, allowing us to determine the most suitable height value for achieving the desired outcomes across all six colors.

**Figure 7:**
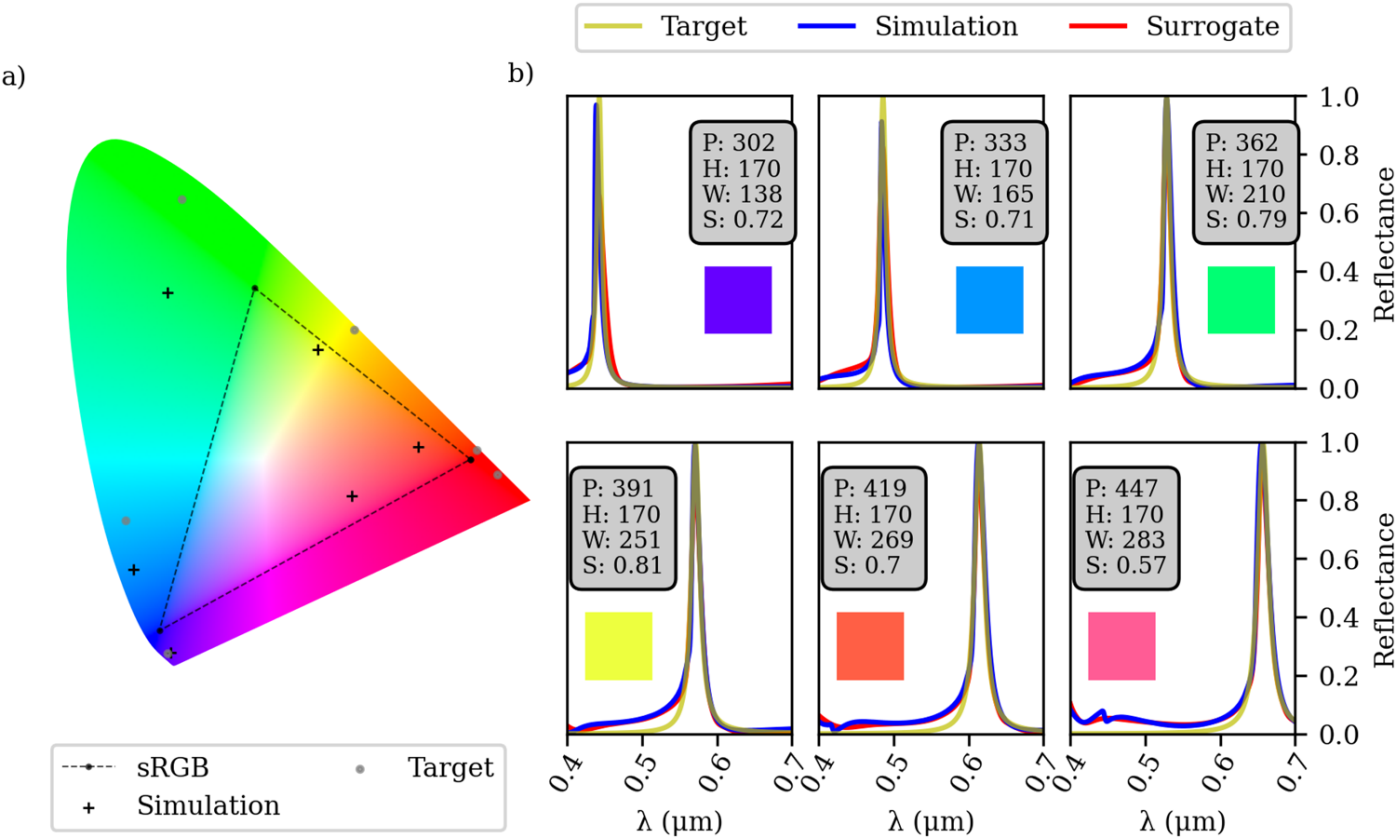
Optimization results based on a fixed height configurations. (**a**) refers to the chromaticity diagram of 6 optimized designs. The simulated spectra is given in (**b**) and the time used for training, and optimizing is depicted in the supplementary information section.

**Figure 8:**
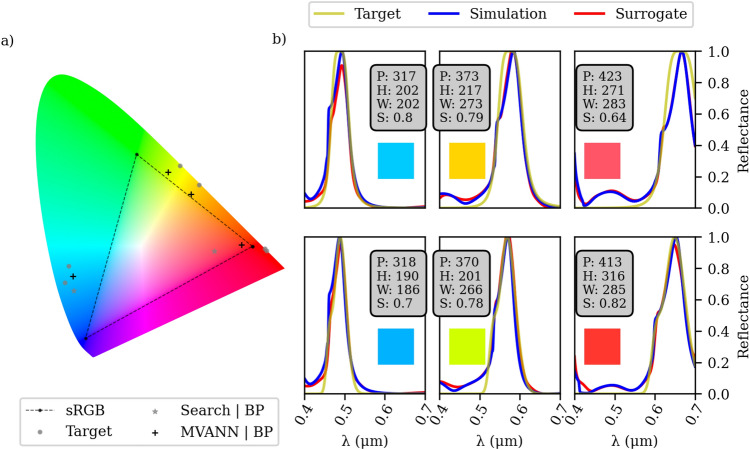
Optimization results targeting a broader Lorentzian ($\omega ^2/(\omega ^2 + 4\left( f - f_0\right) ^6)$) on the top row and a combination of Gaussians to mimic a strong secondary resonance on the bottom row. (**a**) Refers to the chromaticity diagram of 6 optimized designs. The simulated spectra is given in (**b**). It can be seen that we achieved the most vivid red.

The results presented in Fig. 7 demonstrate the effectiveness of our approach in achieving favorable outcomes for most of the considered colors. Our optimization approach allows us to explore various line shapes using the same dataset, as depicted in Fig. 8. Notably, our algorithm excels at identifying optimized designs that seamlessly match the desired response. It is important to mention that our study focuses on achieving pure colors across the entire spectrum, rather than emphasizing a single color. Yet, interestingly, our algorithm is capable of optimizing designs to replicate a vibrant red color response, a task that traditionally involves complex optimization processes (see^20,44^). However, our design approach, coupled with advanced inverse design techniques, simplifies the process of identifying designs that mimic this vivid red color response, all while relying on the same dataset. Furthermore, it is worth noting that prior studies employed half the number of simulations to optimize a single color compared to our methodology.

## Conclusion

In conclusion, our research presents a novel DL methodology for optimizing resilient designs of vivid color filter metasurfaces. By utilizing a surrogate model constructed from a dataset of only 585 simulations, our approach demonstrates exceptional efficiency in the optimization process. The numerical tool developed in this study enables the cost-effective fabrication of structural color filters by exploring a wide range of narrow line shapes that exhibit high-quality resonances, aligning with desired spectral reflection responses. Notably, our methodology expands the color gamut beyond the conventional RGB colors, offering unprecedented versatility in color generation. Furthermore, our DL approach successfully respects fabrication constraints, ensuring practical feasibility. The achievements of our research significantly contribute to the field of optical device design. By pushing the boundaries of metasurface optimization, we open up new possibilities for the development of advanced optical devices. The proposed methodology holds promise for various applications, such as display technologies, data encoding, and artistic expression. These notable advancements not only enhance the understanding of metasurface design principles but also provide valuable insights for future research endeavors.

### Supplementary Information

Supplementary Information 1.

## Data Availability

The data that support the findings of this study are available from the corresponding author upon reasonable request.

## References

[1] Bohren CF, Huffman DR (2008). Absorption and Scattering of Light by Small Particles.

[2] Kerker M (2016). The Scattering of Light and Other Electromagnetic Radiation.

[3] Brown MA, De Vito SC (1993). Predicting azo dye toxicity. Crit. Rev. Environ. Sci. Technol..

[4] Kim H (2009). Structural colour printing using a magnetically tunable and lithographically fixable photonic crystal. Nat. Photonics.

[5] Chen WT (2014). High-efficiency broadband meta-hologram with polarization-controlled dual images. Nano Lett..

[6] Hu H, Chen Q-W, Tang J, Hu X-Y, Zhou X-H (2012). Photonic anti-counterfeiting using structural colors derived from magnetic-responsive photonic crystals with double photonic bandgap heterostructures. J. Mater. Chem..

[7] Ding F, Pors A, Bozhevolnyi SI (2017). Gradient metasurfaces: A review of fundamentals and applications. Rep. Prog. Phys..

[8] Khaidarov E (2022). Large-scale vivid metasurface color printing using advanced 12-in. immersion photolithography. Sci. Rep..

[9] Kuznetsov AI, Miroshnichenko AE, Brongersma ML, Kivshar YS, Luk’yanchuk B (2016). Optically resonant dielectric nanostructures. Science.

[10] Zheng G (2015). Metasurface holograms reaching 80% efficiency. Nat. Nanotechnol..

[11] Arbabi E, Kamali SM, Arbabi A, Faraon A (2018). Full-stokes imaging polarimetry using dielectric metasurfaces. ACS Photonics.

[12] Tittl A (2018). Imaging-based molecular barcoding with pixelated dielectric metasurfaces. Science.

[13] Yang Y, Kravchenko II, Briggs DP, Valentine J (2014). All-dielectric metasurface analogue of electromagnetically induced transparency. Nat. Commun..

[14] Arbabi A, Horie Y, Bagheri M, Faraon A (2015). Dielectric metasurfaces for complete control of phase and polarization with subwavelength spatial resolution and high transmission. Nat. Nanotechnol..

[15] Shalaev MI (2015). High-efficiency all-dielectric metasurfaces for ultracompact beam manipulation in transmission mode. Nano Lett..

[16] Lin D, Fan P, Hasman E, Brongersma ML (2014). Dielectric gradient metasurface optical elements. Science.

[17] Decker M (2015). High-efficiency dielectric huygens’ surfaces. Adv. Opt. Mater..

[18] Yu YF (2015). High-transmission dielectric metasurface with 2$$\pi $$ phase control at visible wavelengths. Laser Photonics Rev..

[19] Um H-D (2023). Dynamic selection of visible wavelengths using resonant TiO_2_ nanostructures. Nanophotonics.

[20] Lin, R. *et al.* An efficient neural optimizer for resonant nanostructures: Demonstration of highly-saturated red silicon structural color. arXiv:2304.13516 (2023).

[21] Shang, X. *et al.* Polarization-sensitive structural colors based on anisotropic silicon metasurfaces. In *Photonics*, vol. 10, 448 (MDPI, 2023).

[22] Ma W, Cheng F, Xu Y, Wen Q, Liu Y (2019). Probabilistic representation and inverse design of metamaterials based on a deep generative model with semi-supervised learning strategy. Adv. Mater..

[23] Peurifoy J (2018). Nanophotonic particle simulation and inverse design using artificial neural networks. Sci. Adv..

[24] Asano T, Noda S (2018). Optimization of photonic crystal nanocavities based on deep learning. Opt. Express.

[25] Jiang J, Fan JA (2019). Global optimization of dielectric metasurfaces using a physics-driven neural network. Nano Lett..

[26] Wen F, Jiang J, Fan JA (2020). Robust freeform metasurface design based on progressively growing generative networks. ACS Photonics.

[27] Wiecha PR, Muskens OL (2019). Deep learning meets nanophotonics: A generalized accurate predictor for near fields and far fields of arbitrary 3d nanostructures. Nano Lett..

[28] Wiecha PR, Arbouet A, Girard C, Muskens OL (2021). Deep learning in nano-photonics: Inverse design and beyond. Photonics Res..

[29] Hemmatyar O, Abdollahramezani S, Kiarashinejad Y, Zandehshahvar M, Adibi A (2019). Full color generation with fano-type resonant hfo 2 nanopillars designed by a deep-learning approach. Nanoscale.

[30] Elsawy MM (2019). Global optimization of metasurface designs using statistical learning methods. Sci. Rep..

[31] Xu D (2021). Efficient design of a dielectric metasurface with transfer learning and genetic algorithm. Opt. Mater. Express.

[32] Jafar-Zanjani S, Inampudi S, Mosallaei H (2018). Adaptive genetic algorithm for optical metasurfaces design. Sci. Rep..

[33] Zhang C, Jin J, Na W, Zhang Q-J, Yu M (2018). Multivalued neural network inverse modeling and applications to microwave filters. IEEE Trans. Microw. Theory Tech..

[34] Liu H (2022). Transfer printing of solution-processed 3d ZnO nanostructures with ultra-high yield for flexible metasurface color filter. Adv. Mater. Interfaces.

[35] Panda SS, Hegde RS (2020). Transmission-mode all-dielectric metasurface color filter arrays designed by evolutionary search. J. Nanophotonics.

[36] Wang Y, Huang W, Lin Y-S, Yang B-R (2022). A tunable color filter using a hybrid metasurface composed of ZnO nanopillars and Ag nanoholes. Nanoscale Adv..

[37] Barton D, Lawrence M, Dionne J (2021). Wavefront shaping and modulation with resonant electro-optic phase gradient metasurfaces. Appl. Phys. Lett..

[38] Lawrence M (2020). High quality factor phase gradient metasurfaces. Nat. Nanotechnol..

[39] Hendrycks, D. & Gimpel, K. Gaussian error linear units (gelus). arXiv:1606.08415 (2016).

[40] Glorot, X. & Bengio, Y. Understanding the difficulty of training deep feedforward neural networks. In *Proceedings of the Thirteenth International Conference on Artificial Intelligence and Statistics*, 249–256 (JMLR Workshop and Conference Proceedings, 2010).

[41] Abadi, M. *et al.* Tensorflow: A system for large-scale machine learning. In *Osdi*, vol. 16, 265–283 (Savannah, 2016).

[42] Kingma, D. P. & Ba, J. Adam: A method for stochastic optimization. arXiv:1412.6980 (2014).

[43] Schanda J (2007). Colorimetry: Understanding the CIE System.

[44] Dong Z (2022). Schrödinger’s red pixel by quasi-bound-states-in-the-continuum. Sci. Adv..

