# Author Correction: Back-propagation optimization and multi-valued artificial neural networks for highly vivid structural color filter metasurfaces

**DOI:** 10.1038/s41598-024-52554-x

**Published:** 2024-01-23

**Authors:** Arthur Clini de Souza, Stéphane Lanteri, Hugo Enrique Hernández-Figueroa, Marco Abbarchi, David Grosso, Badre Kerzabi, Mahmoud Elsawy

**Affiliations:** 1 Université Côte d’Azur, Inria, CNRS, LJAD, 06902 Sophia Antipolis Cedex, France; 2 Laboratory of Applied and Computational Electromagnetism (LEMAC), School of Electrical and Computer Engineering (FEEC), University of Campinas (UNICAMP) Campinas, São Paulo, Brazil; 3 Solnil, 95 Rue de la République, 13002 Marseille, France; 4 Université Aix Marseille, CNRS, Université de Toulon, IM2NP, UMR 7334, F-13397 Marseille, France

Correction to: *Scientific Reports* 10.1038/s41598-023-48064-x, published online 04 December 2023

The original version of this Article contained an error in the spelling of the author Hugo Enrique Hernández-Figueroa, which was incorrectly given as Hugo Enirique Hernández-Figueroa.

The original Article has been corrected.

