# Supplementary material for: Back-propagation optimization and multi-valued artificial neural networks for highly vivid structural color filter metasurfaces
Source: Sci Rep. 2023 Dec 4;13:21352. doi: 10.1038/s41598-023-48064-x (PMC10695957; doi:10.1038/s41598-023-48064-x)
Supplement: Supplementary file 1 — Supplementary Information 1. [file 41598_2023_48064_MOESM1_ESM.pdf]

Supplementary information

1. SIMULATION SETUP

The simulations are realized using python's MEEP library [1].The default resolution is 150 pixels per µm, and therefore, 105 pixels per longest wavelength. However, if the cell size of the simulation is not an integer number of pixels, instead of adapting the resolution, the default MEEP procedure is to change the cell size. In our case, we require fine control over the period. That is why we instead used a value close to 150 nm that resulted in the cell dimensions to be an integer number of pixels. MEEP stills warns that the grid volume is not an integer number of pixels and changes slightly the cell size due to the truncation of some values. Each simulation only evaluates a single resonator and there is periodic boundary condition on each boundary of the simulation. Perfectly Matched Layer (PML) layers with 0.8 µm length were positioned on the extremes of the simulation except from the sides. MEEP is known to have issues regarding periodic boundary conditions and PMLs, and the longer the interface between them, the more artifacts the simulation acquires and easier it becomes to diverge. The other option was using Absorber layers, the problem is that they require much more space to fully extinguish the fields and increases the simulation time. Some reaching 20 minutes. As the length of the PML is slightly larger than the longest wavelength, it extinguishes the fields while having imperceptible simulation artifacts. The same simulation that would last 20 minutes with the absorber layer, now takes only 14 seconds. The total height of the simulation is 3.1 µm. The height of the cell is adjusted if the resolution is updated. The light is injected from top with normal incidence with electric field polarized in the x-direction. There are 5 uniformly distributed in frequency Gaussian sources to guarantee that floating point errors does not interfere with the results.The transmission spectrum was sampled from 400 nm to 700 nm with 500 points on the bottom of the simulation. An electric filed sampling on the x-direction was positioned between the sources and the middle of the simulation. It records the maximum value it ever encounters and then it stops the simulation if the field recorded on that point is 10^{-8} smaller than the maximum value it found. It then performs a Fast Fourier Transform (FFT) to compute the transmission spectrum. As there are no losses in the simulation, the reflection spectrum is calculated using the transmission spectrum. In order to calculate the transmission spectrum, firstly it is required to validate the "raw" transmission without any structure. Therefore, computing the spectrum of the source. By varying the period of the resonator, we observe changes the intensity of the transmission as seen in Figure S1 a).

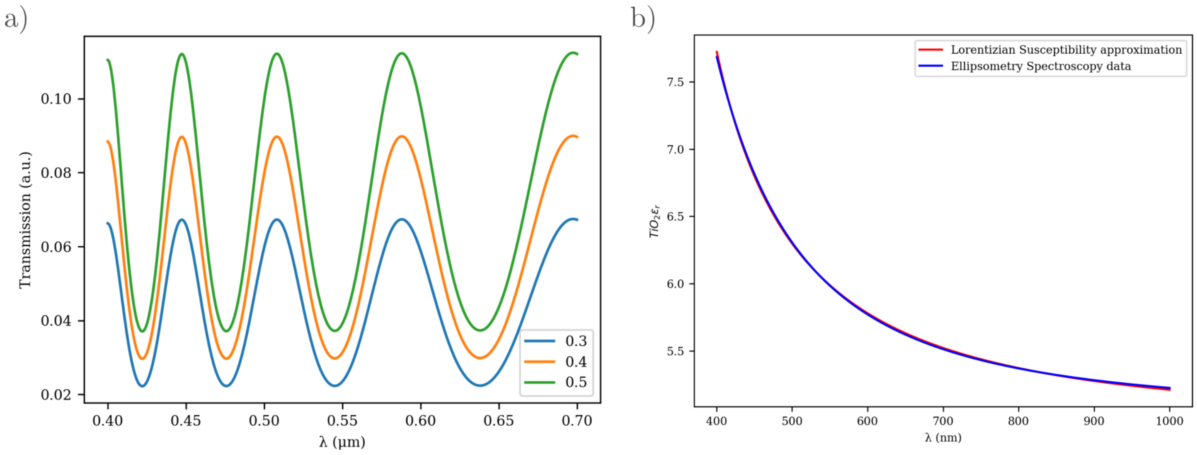

Fig. S1. a) Transmission spectrum without any structure. Each curve represent a different resonator period. b) Comparative analysis of the measured dispersion of TiO_2 and the approximation generated through modeling using MEEP. The experimental data capturing the dispersion characteristics were obtained through Ellipsometry spectroscopy detailed below. Subsequently, the collected data were fitted to a Lorentzian susceptibility function. Notably, the approximation achieved through the modeling process closely aligns with the measured dispersion, demonstrating the efficacy of the approach in accurately representing the dispersion behavior of TiO_2

TiO_2 Ellipsometry Spectroscopy

The characterization of the two-dimensional flat layers made of TiO_2 was performed in order to determine their optical constants. The measurements were carried out with a Wollam M200V ellipsometer (350-1000 nm) on a layer obtained via sol-gel chemistry and dip-coating on a bulk Si substrate[2]. The ellipsometric data (Δ and Ψ) are fitted by a Cauchy model to extract refractive index n and extinction coefficient k (being the latter equal to zero in the considered wavelength range). These layers are of optical quality being their surface roughness below 0.1 nm [3].

Measuring the refractive index evolution of a TiO_2 thin layer kept in a ellipsometry-porosimetry chamber with controlled humidity (atmosphere where air and water were progressively mixed) we assess the adsorption/desorption of water. From the corresponding isotherms we obtain the mesoporosity of the layers[4] (pore volume, size distribution, and interconnection through adapted Kelvin models). Measurements on dense TiO_2 (n > 2.4) account for a porosity well below 10%

The refractive index of the TiO_2 was fitted by a Lorentzian susceptibility function using scipy's optimize curve_fit function with initial guess 1.1 for epsilon, 3.85 of frequency and 3.75 of σ. The final parameters values are 1.87681265 for epsilon, 3.63269272 for frequency and 3.08109585 for σ. The difference between the ellipsometry data and the fitted curve can be seen in Figure S1 b).

To calculate the spectrum source for every period, we assumed a linear correlation between the period and the magnitude of the transmittance, and performing a linear interpolation for every point in the spectrum, obtaining the source vector **a**. The linear coefficient was close to 0 for all 500 points, and therefore, considered to be exactly 0. And therefore, to obtain the source spectrum of a resonator with period P, we can simply multiply the source vector **a** with the period of the metasurface.

A comparison of MEEP Finite-Difference Time-Domain (FDTD) simulation and COMSOL multiphysics Finite Element Method (FEM), has been and and shows on Figure S2 a) and b).

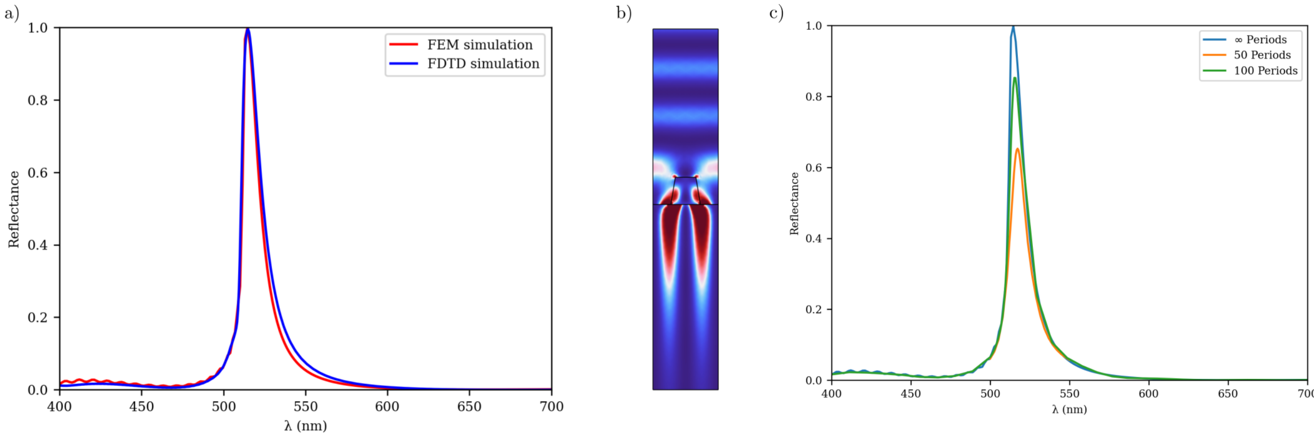

Fig. S2. Comparison between the FDTD MEEP simulations and FEM COMSOL simulations. With P = 353 nm, H = 147 nm, W = 159 nm, S = 0.3. a) Reflection spectrum and b) Electric field normalized at the wavelength corresponding to the peak reflactance. c) Difference between different array sizes.

Figure S2 c) shows how the reflectance, transmittance and loss responds to different array sizes. As the period is 353 nm, the last configuration with 100 repetitions is 35.3 µm in length. And as seen, the resonance requires many repetitions to achieve the desired performance. The reason for this behavior is the mode excited by the resonant frequency. Which propagates in the x-axis in the substrate as seen by Figure S3.

2. SURROGATE TRAINING

Figure S4 displays the loss evolution at each epoch.

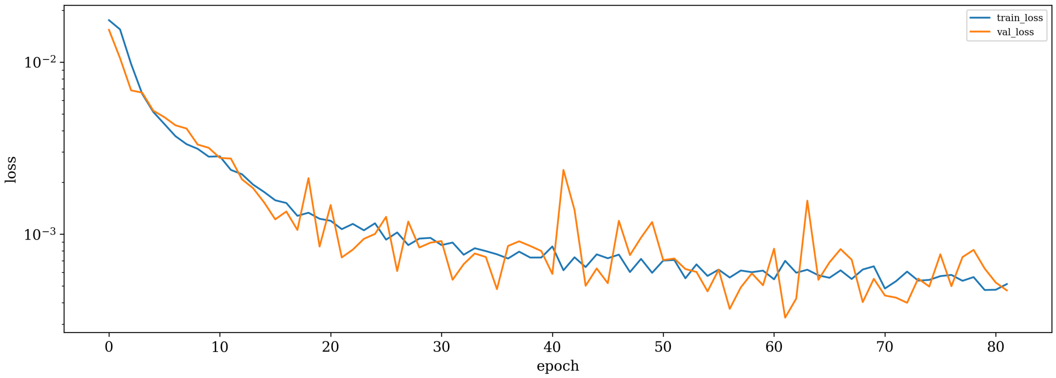

Fig. S4. Mean Square Error (MSE) loss of the training and validation sets in function of the epoch for the surrogate model.

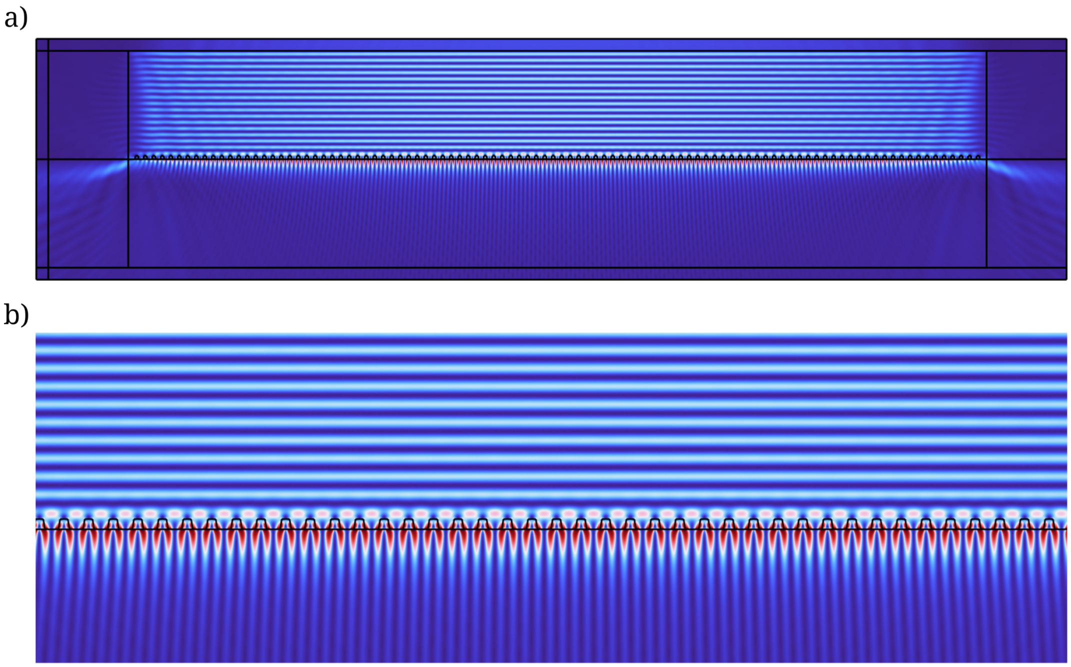

Fig. S3. Fields computed at the wavelength of 515 nm for the scenario of 100 repetitions. a) Electric field normalized of the entire simulation domain. b) Zoom at the center of the simulation domain to obtain a better view of the local fields near the resonators.

3. BACK-PROPAGATION OPTIMIZATION METHODOLOGY

By disabling the training of the weights of the surrogate model and added one layer before the input. The added layer has 5 inputs and the weights were initialized with the identity. The 4 first inputs (geometry parameters) are fully connected with the first 4 inputs of the surrogate model and the last input (λ) is solely connected to the surrogate model input referent to the wavelength. The training of this connection is disabled. The activation function for the described layer was Rectified Linear Unit (ReLU) to prevent negative values. The most important part is the training. We used full batch Adam optimizer with initial learning rate of 10^{-5} with exponential decay with 1000 decay steps and decay rate of 0.98. These choices proved necessary due to the needed small adjustments and the gradients grow after passing through the entire Artificial Neural Network (ANN) without any Batch Normalization (BN) layer. The training spanned 1800 epochs with patience of 6 with minimum delta of 10^{-6}, meaning if the loss decrease less than 10^{-6} in 6 epochs, the training stops. We present the Python code using tensorflow and keras framework to run the back-propagation optimization.

```
lr_schedule = tf.keras.optimizers.schedules.ExponentialDecay(initial_learning_rate=1e-5,
                                                  decay_steps=1000,
                                                  decay_rate=0.98)  # Learning rate schedule
opt = tf.keras.optimizers.Adam(learning_rate=lr_schedule)   # Using Adam

# Patience
callback = tf.keras.callbacks.EarlyStopping(monitor='loss', patience=6, min_delta=1e-6)

# Define the model to be trained
Input_all = Input(shape=(5,))   # Input that will receive the initial guess

# Layer responsible for finding the geometrical parameters.
d1 = Dense(4, kernel_initializer='identity', activation='relu')(Input_all[:,:-1])

# Layer that will pass the wavelength. Note that we do not want to optimize this layer.
d2 = Dense(1, kernel_initializer='identity', trainable=False)(Input_all[:,-1:])

d3 = Concatenate()([d1,d2])
inverse_designer = Model(inputs=Input_all, outputs=d3)

Surrogate_model.trainable = False   # Freeze the weight of the surrogate model.
Full_model_outputs = Surrogate_model(inverse_designer.output)

# Creating the model comprising the surrogate and our layer
Full_model = Model(inverse_designer.input, Full_model_outputs)
Full_model.compile(optimizer=opt, loss='mse')

# Initial guess here can be from the MVANN or from the Search
```

```python
# Its simply a list in the following format: [P, H, W, S]
ipt_i_tolist = initial_guess.tolist()

# We then create a dataset comprising the combination of  same input and all the wavelengths
# wl is a list of all wavelengths
eng_inp = np.array([ipt_i_tolist + [w] for w in wl])

# The next step is training the new layer. Note that we want full batch optimization
# t_f is the target response
history = Full_model.fit(eng_inp, np.array(t_f), epochs=1800, callbacks=[callback],
                        verbose=0, batch_size=500)

# Then, to obtain the optimized result, we call it.
bp_result = inverse_designer.predict(eng_inp, verbose=0)
```

We decided to incorporate an additional layer for optimization rather than directly computing and applying gradients to the design space. This decision was primarily driven by the fact that the framework already had features like early stopping and learning rate schedules in place, which we found beneficial for our approach.

4. OPTIMIZATION FOR LOWER QUALITY FACTOR RESONANCE TARGETS

By performing the dataset search with relaxed objectives that concerns a broader Full Width at Half Maximum (FWHM), a reasonable solution can be found on the dataset. Turning the Remaining optimization methodology unnecessary as seen by Figure S5.

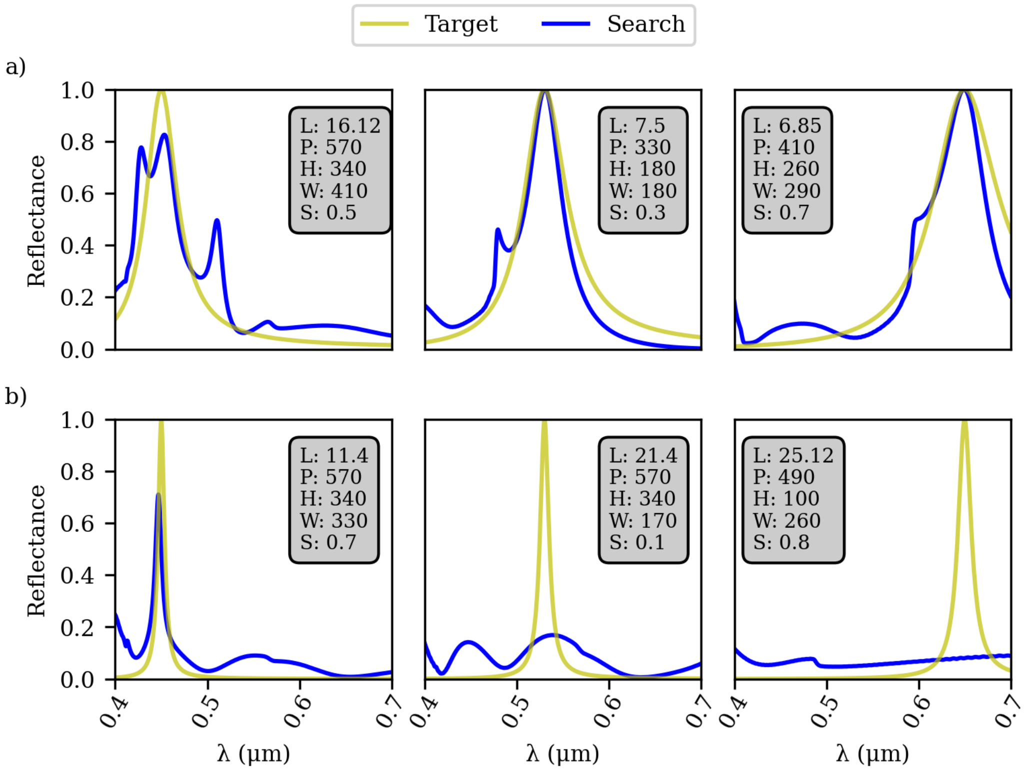

Fig. S5. Results from the dataset search using a Lorentzian lineshape in frequency as target for the dataset search for the wavelengths of 450 nm, 560 nm and 650 nm with a) $\omega = 0.05$ and b) $\omega = 0.01$. L is the MSE between the target spectrum and the closest one in the dataset, and P, H, W and S are the geometric parameters. As seen, unlike the low quality target objectives give in (a), the dataset search could not find a good solution when targeting sharp resonances as it is depicted in b).

As our objective is relaxed, there is no need to refine the solutions found by searching the dataset.

5. MVANN

A Multi-Valued Artificial Neural Network (MVANN) is simply a ANN capable of outputting multiple solutions given a single input. The adoption of the MVANN in this context is particularly advantageous due to its ability to mitigate the issue of

multiple parameter responses for a given objective target [5]. This feature ensures that the MVANN provides reliable and consistent results in non-unique scenarios where multiple solutions can arise from a single input target spectrum.

One of the greatest challenges even for a MVANN is the fact that we are extrapolating, because the data that is going to be used after training is different from the data used to train it. Although looking similar to some training data, a pure Lorentzian curve does not exist in the simulated data, and a small difference between it and a pure Lorentzian spectrum can cause the ANN to not generalize well, because ANN are used to interpolate functions and therefore, should not be applied to extrapolation [6]. There are several solutions to this problem. One of them consists in having a Deep Learning (DL) model capable of extrapolating well. One such example are Physics-Informed Neural Networks (PINNs) that hard codes the physics of the problem into the ANN and can extrapolate extremely well [7]. The problem for our case is that we cannot extract simple differential equations that correlates the input to the output, and therefore, PINNs cannot be considered. The last solution and the least efficient is to apply regularization to the training parameters. By doing so, we manipulate the bias-variance trade off, and by having a lower variance model with higher bias our ANN is able to extrapolate with the downside of losing interpolation performance.

Matching the correct output given a spectrum becomes an arduous task, as it necessitates accurately aligning the corresponding parameters across all wavelengths encompassed within the spectrum. Training with only 810 data points is not enough to obtain reasonable results. A solution is to generate data with the surrogate model to be used as training for the MVANN. We generated 7839 input-output pairs in 4 min and 13 seconds on a Google colab's Central Processing Unit (CPU). The geometry limits are the same as for the simulations, the only difference is the step, which now is 25 nm instead of 65 nm.

The architecture of the MVANN is represented by Figure S6. We opted to use residual blocks as they usually grant us good generalization with a reasonable depth [8, 9]. On top of that, we also used L_1 regularization, by summing all the weights on the network, and multiplying by the penalization strength, on this case, 10^{-7}, as the ANN had 2 023 808 trainable parameters, meaning an average penalization of 0.2 per parameter. All activation functions were Gaussian Error Linear Unit (GELU) except for the last layers of the output block and the kernel initialiser was Glorot normal for all parameters [10, 11]. BN Layers and dropout layers with high rate were employed to enhance the generalization and extrapolation capacity of the ANN.

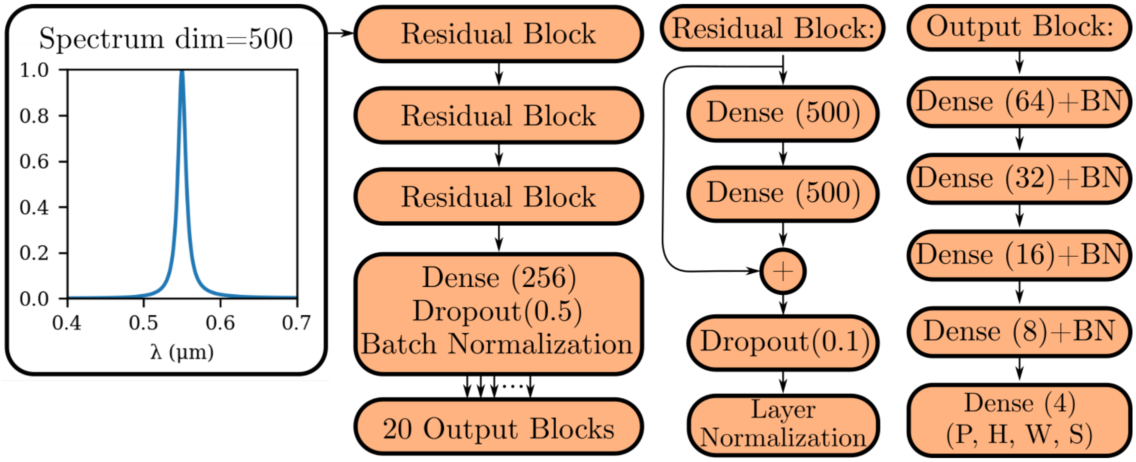

Fig. S6. Schematic of the MVANN Architecture. We used 3 residual blocks with 500 neurons per layer followed by a layer with 256 neurons, a dropout layer with rate = 0.5 and batch normalization. Each output block consists of 5 layers, each followed by a batch normalization layer. The first layer starts with 64 neurons and geometrically decays to 4.

The model was trained with tensorflow for 100 epochs with patience of 10 [12]. However, differently from the surrogate model, we have not used holdout.

The decision to exclude the use of holdout training for the MVANN may be viewed as controversial, but it yielded superior initial estimates for the subsequent back-propagation optimization process. Identifying concrete reasons for this phenomenon can be challenging, as it involves various factors, such as the small size of the dataset used for training, the potentially inadequate number of outputs, or the inherent nature of the MVANN in requiring a vast amount of training data for optimal performance in this particular scenario. Additionally, the notion of withholding data for assessing overfitting becomes less crucial in this context, as each additional data point could provide relevant information due to the high sensitivity of the dataset. It is important to note that the results obtained using the MVANN are going to be used as initial guesses for back-propagation optimization and do not necessitate perfection.

The chosen optimizer is Adam and the batch size has been fixed to 32 [13]. The training stopped at epoch 100 with final loss of 2.3093×10^{-4}. The total training time was 46 min 8 s on a google colab's CPU.

The loss function of the MVANN illustrates a crucial characteristic of the behaviour of the model. This being said, that the presence of a single low loss output solution has a significant impact on the final loss value. This implies that the attainment of a single high-quality solution effectively determines the calculated loss, thereby compelling the specialization of each individual

output. Subsequently, we exploit the advantageous properties of the surrogate model ANN, which is fully differentiable. This enables us to propagate gradients through the model using back-propagation, allowing for efficient refinement of the solutions initially provided by the MVANN. For this specific study, we employed a configuration with 20 outputs, which proved to be sufficient in offering reasonable starting guesses for the back-propagation optimization. The loss function for the MVANN is explained in the original material [5]. A small modification of the loss function is used in this study and displayed on the supplementary information section S2.

To quantify the performance of the MVANN, we calculated the loss function $L(\mathbf{w})$ over a batch of size N, as represented by Equation (S1).

$$L(\mathbf{w}) = \frac{1}{N}\sum_{i=1}^{N}\frac{1}{B_i(\mathbf{w})} \tag{S1}$$

The weights $\mathbf{w}$ are involved in the calculation of the loss, and $B_i(\mathbf{w})$ represents the inverse of the loss for a single batch, defined as the sum of the inverse losses for each output, as:

$$B_i(\mathbf{w}) = \sum_{j=1}^{N_{outs}}\frac{1}{S_j(\mathbf{w})} \tag{S2}$$

Here $S_j(\mathbf{w})$ is the L_2 distance between the output j of the MVANN, $\mathbf{G}_{\text{ANN},j}(\mathbf{x},\mathbf{w})$ given the input $\mathbf{x}$ and the desired response $\mathbf{d}$,

$$S_j(\mathbf{w}) = ||\mathbf{G}_{\text{ANN},j}(\mathbf{x},\mathbf{w}) - \mathbf{d}||_2^2 \tag{S3}$$

The motivation for the utilization of such loss function is to lower the losses for conflicting outputs and is explained in the original material [5]. However, we give here a quick explanation If a single output matches very well, $S_j(\mathbf{w}) \to 0 \implies B_i(\mathbf{w}) \to \infty$ which will imply that the loss for that point of data is going to tend to 0 even if there is a $S_j(\mathbf{w}) \to \infty$ [5]. The only difference between the original MVANN and the used on this paper is that we haven't divided by 2 the L_2 distance represented by Equation (S3) [5].

Figure S7 displays the loss evolution at each epoch. The validation loss considers the same dataset used to train it, and it is lower than the train loss due to the use of dropout, batch normalization layers and most importantly, the L1 regularization term that is only present in the training loss and not in the validation.

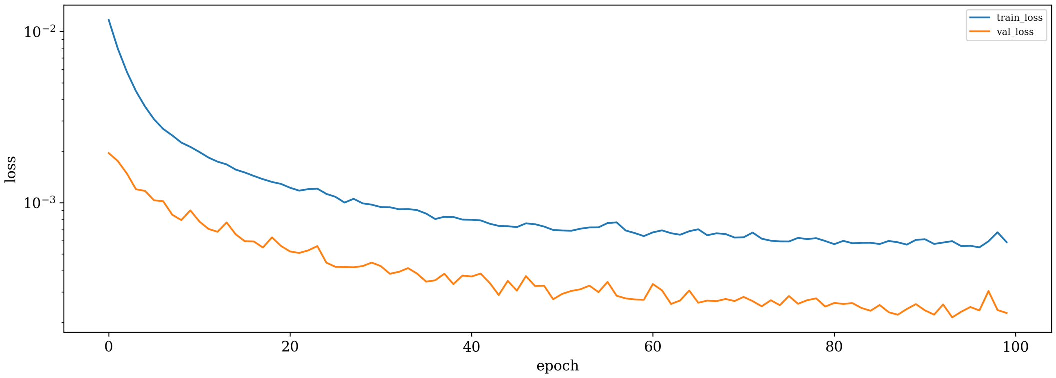

Fig. S7. MSE loss of the training and validation sets in function of the epoch for the MVANN model.

6. STUDY OF THE EFFICACY OF THE MVANN WITH DIFFERENT DATASET SIZES

In this section, we investigate how the dataset size affects the performance of the MVANN. In Figure S8, we present the MSE between the target function and the optimized design at various wavelengths. The orange curve corresponds to the search with back-propagation, while the blue curve represents the MVANN with back-propagation for four different dataset sizes. Notably, the MVANN with back-propagation consistently outperforms the search method. The results from the 585 simulations demonstrate the lowest MSE for the MVANN with back-propagation. This case, involving 585 simulations, essentially represents the limit beyond which further dataset size increases do not yield performance improvements. In other words, increasing the dataset size beyond this point does not enhance performance. Also, it can be observed that it is more common for the MVANN to outperform the search.

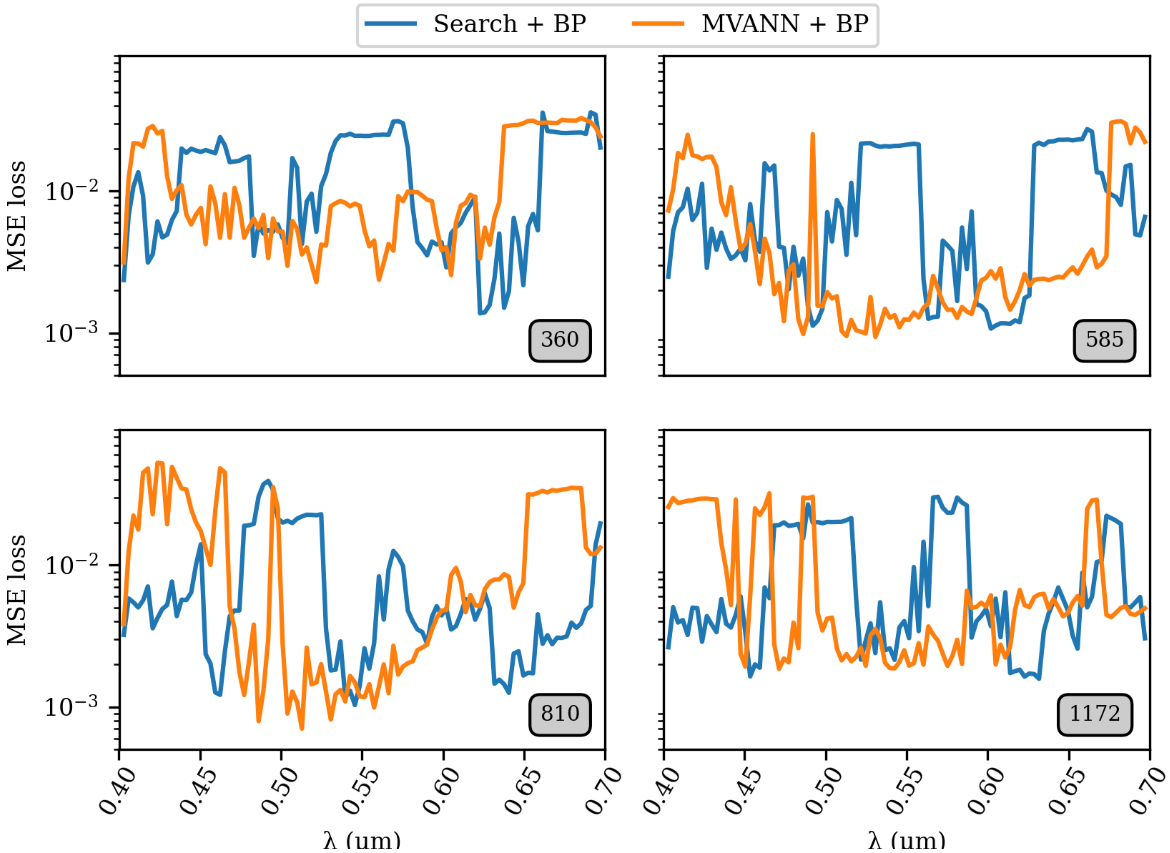

Fig. S8. MSE between target function and optimized design at each wavelength. The search scan with back-propagation is represented by the orange curve, while and the MVANN with back-propagation is given by the blue curve for 4 different dataset sizes.

7. COMPARISON BETWEEN MVANN AND STATE-OF-THE-ART ALGORITHMS

In Figure S9, we compared the MVANN with a β conditional Variational Autoencoder (β-cVAE) [14, 15]. The β-cVAE model utilized a similar architecture to the MVANN, comprising 3 645 288 trainable parameters and having 2 latent dimensions. Training was conducted over 100 epochs using AdamW with a learning rate of 3×10^{-4} and MSE loss. The training process took 41 minutes and 40 seconds. For comparison, training the MVANN on the same CPU typically requires around 50 minutes. The results can be seen in Figure S9. One of the challenges associated with the β-cVAE is that it necessitates an optimization technique for determining the latent variables, unlike the MVANN where the solution is typically present in at least one of the outputs. In our case, we applied a brute-force optimization by randomly guessing 20 values to make the comparison with the MVANN more equitable. Furthermore, similar to the MVANN, we employed back-propagation optimization for the design variables. The β-cVAE encoder consists of a first layer with 500 neurons, followed by 3 residual layers, and then 128 neurons. The decoder follows the same architecture, with the addition of a layer with 4 neurons after the one with 128 neurons. All layers utilize GELU activation with Glorot normal initialization, except for the layers responsible for computing the mean and the logarithm of the variance before the sampling layer. In conclusion, as demonstrated in Figure S9, both methods generally exhibit similar performance. However, the β-cVAE has two notable drawbacks when compared to the MVANN. Firstly, it still requires an optimization technique to find the latent variables, while in the MVANN, the solution is typically present in at least one of the outputs. Secondly, the implementation of the β-cVAE is less straightforward and more convoluted compared to the MVANN.

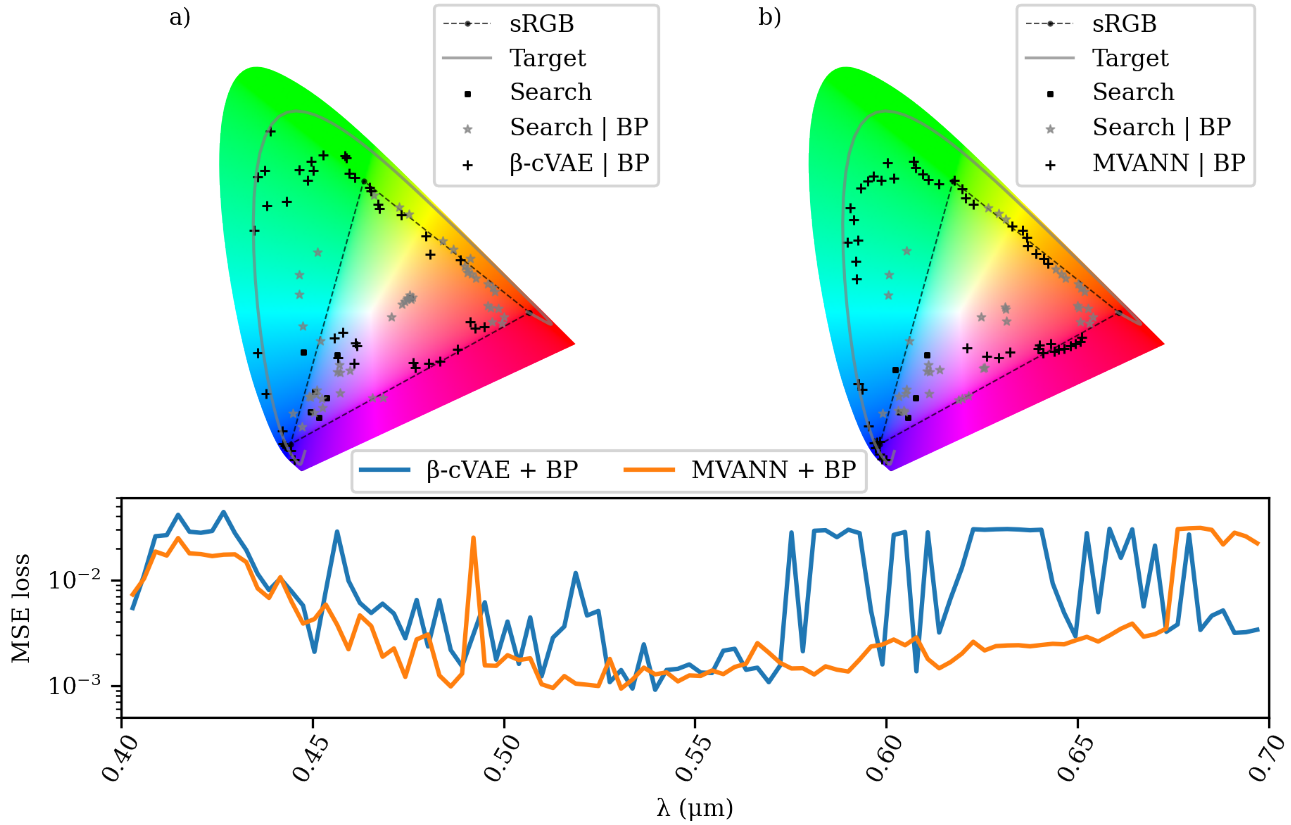

Fig. S9. Chromaticity diagram and MSE loss comparing the target with the simulated spectrum of the optimized design for the a) β-cVAE and b) MVANN after back-propagation for a dataset of size 585. They exhibit similar performance in terms of MSE and the chromaticity diagram, except for targets above 575 nm.

One metric to benchmark the MVANN and the Search after back-propagation is to compare the average MSE and standard deviation. However, it does not tell the whole story, as it is observed a bi-stable regime, where it oscillates between two values, and therefore, the minimum and maximum values are also taken into account. Table S1 benchmarks the different dataset sizes.

Table S1. Table representing the mean, standard deviation (σ), minimum and maximum values for the MSE loss between the simulated and target spectrum for different dataset sizes and methods. The values are multiplied by 10^3.

Values in 10^{-3}		Mean	σ	min	max
360	MVANN + BP	12.8	10.37	2.28	32.82
	Search + BP	13.92	9.8	1.37	35.96
585	MVANN + BP	6.19	8.26	0.94	31.16
	Search + BP	9.65	8.23	1.07	27.40
	β-cVAE + BP	12.06	11.98	0.91	44.14
810	MVANN + BP	13.78	15.15	0.70	52.74
	Search + BP	7.94	8.42	1.03	39.30
	β-cVAE + BP	16.1	15.51	0.81	56.86
1172	MVANN + BP	9.37	10.26	1.86	32.15
	Search + BP	9.55	8.53	1.58	30.37

It can be seen that the best case scenario is the dataset with 585 simulations, showing the MVANN after back-propagation to have the least mean, however, for 810, the minimum is lower, and considering Figure S8, that minimum lasts from 475 nm to 575 nm, which is mostly the whole green and cyan areas. However, this region exhibits instability and spikes. Other conclusion is the whole stability of the MVANN with back-propagation in the scenario with 585, outperforming the search in multiple areas.

8. TIME TABLE

Table S2. Time required for various stages, including data generation, model training, and optimization runs. The simulations were conducted on the Google Colab platform equipped with an Intel® Xeon® E5 v4 Processor operating at 2.2 GHz. It should be noted that determining the optimization time per design in the fixed height case can be misleading, as the back-propagation time does not scale linearly with the number of optimizations due to training on the same batch. Furthermore, the time needed to identify designs with the closest heights exhibits a significant increase as the number of simultaneous optimizations rises. For instance, while 6 designs required a mere 0.8 seconds, this duration is extended to 5 minutes and 30 seconds when dealing with 10 designs.

Simulations		Models Training		Free height optimization (1 design)		Fixed height optimization (6 designs)	
Total	33 min 40 s	Surrogate	9 min 48 s	Simulations	51.8 s	Simulations	2 min 57 s
Average	3.4 s	Generation	4 min 13 s	Back-propagation	37.3 s	Back-propagation	1 min 18 s
		MVANN	46 min 8 s	Predictions	5.1 s	Predictions	15 s
		Total	1h 0 min 9 s	Search	2.5 ms	Total	4 min 30 s
				Total	1 min 34 s	Per design total	35 s

REFERENCES

1. A. F. Oskooi, D. Roundy, M. Ibanescu, P. Bermel, J. D. Joannopoulos, and S. G. Johnson, "Meep: A flexible free-software package for electromagnetic simulations by the fdtd method," Comput. Phys. Commun. **181**, 687–702 (2010).
2. E. Bindini, G. Naudin, M. Faustini, D. Grosso, and C. Boissière, "Critical role of the atmosphere in dip-coating process," The J. Phys. Chem. C **121**, 14572–14580 (2017).
3. Z. Chehadi, M. Bouabdellaoui, M. Modaresialam, T. Bottein, M. Salvalaglio, M. Bollani, D. Grosso, and M. Abbarchi, "Scalable disordered hyperuniform architectures via nanoimprint lithography of metal oxides," ACS Appl. Mater. & Interfaces **13**, 37761–37774 (2021).
4. J. Loizillon, B. Baumgartner, C. Sinturel, M. Abbarchi, B. Lendl, and D. Grosso, "In-depth study of coating multimodal porosity using ellipsometry porosimetry in desorption scanning mode," The J. Phys. Chem. C **123**, 23464–23479 (2019).
5. C. Zhang, J. Jin, W. Na, Q.-J. Zhang, and M. Yu, "Multivalued neural network inverse modeling and applications to microwave filters," IEEE Transactions on Microw. Theory Tech. **66**, 3781–3797 (2018).
6. D. Acharige and E. Johlin, "Machine learning in interpolation and extrapolation for nanophotonic inverse design," ACS omega **7**, 33537–33547 (2022).
7. M. Raissi, P. Perdikaris, and G. E. Karniadakis, "Physics-informed neural networks: A deep learning framework for solving forward and inverse problems involving nonlinear partial differential equations," J. Comput. physics **378**, 686–707 (2019).
8. F. He, T. Liu, and D. Tao, "Why resnet works? residuals generalize," IEEE transactions on neural networks learning systems **31**, 5349–5362 (2020).
9. A. E. Orhan and X. Pitkow, "Skip connections eliminate singularities," arXiv preprint arXiv:1701.09175 (2017).
10. D. Hendrycks and K. Gimpel, "Gaussian error linear units (gelus)," arXiv preprint arXiv:1606.08415 (2016).
11. X. Glorot and Y. Bengio, "Understanding the difficulty of training deep feedforward neural networks," in *Proceedings of the thirteenth international conference on artificial intelligence and statistics,* (JMLR Workshop and Conference Proceedings, 2010), pp. 249–256.
12. M. Abadi, P. Barham, J. Chen, Z. Chen, A. Davis, J. Dean, M. Devin, S. Ghemawat, G. Irving, M. Isard *et al.*, "Tensorflow: a system for large-scale machine learning." in *Osdi,* vol. 16 (Savannah, GA, USA, 2016), pp. 265–283.
13. D. P. Kingma and J. Ba, "Adam: A method for stochastic optimization," arXiv preprint arXiv:1412.6980 (2014).
14. H. Lin, J. Hou, Y. Wang, R. Tang, X. Shi, Y. Tian, W. Xu *et al.*, "Machine-learning-assisted inverse design of scattering enhanced metasurface," Opt. Express **30**, 3076–3088 (2022).
15. R. Yu, Y. Liu, and L. Zhu, "Inverse design of high degree of freedom meta-atoms based on machine learning and genetic algorithm methods," Opt. Express **30**, 35776–35791 (2022).
